# Supplementary material for: Cross-linked polyaniline for production of long lifespan aqueous iron||organic batteries with electrochromic properties
Source: Nat Commun. 2023 May 30;14:3117. doi: 10.1038/s41467-023-38890-y (PMC10229554; doi:10.1038/s41467-023-38890-y)
Supplement: Supplementary file 1 — Supporting Information [file 41467_2023_38890_MOESM1_ESM.pdf]

Supplementary Information

Cross-linked polyaniline for production of long lifespan aqueous iron||organic batteries with electrochromic properties

Haiming Lv[1 2,], Zhiquan Wei,[4,] Cuiping Han[3*], Xiaolong Yang[1], Zijie Tang[1], Yantu Zhang[2], Chunyi, Zhi[1, 4*], Hongfei Li[1,5*]

1 Songshan Lake Materials Laboratory, Dongguan, Guangdong, 523808, China

2 Key Laboratory of New Energy & New Functional Materials, Shaanxi Key Laboratory of Chemical Reaction Engineering, College of Chemistry and Chemical Engineering, Yan'an University, Yan'an, Shaanxi, 716000, PR China

3 Faculty of Materials Science and Engineering, Low Dimensional Energy Materials Research Center, Shenzhen Institutes of Advanced Technology, Chinese Academy of Sciences, Shenzhen, 518055 China

4 Department of Materials Science and Engineering, City University of Hong Kong, Hong Kong, 999077, China

5 School of System Design and Intelligent Manufacturing, Southern University of Science and Technology, Shenzhen, Guangdong 518055, China;

These authors contributed equally: Haiming Lv, Zhiquan Wei

*Corresponding author:

✉ Email: cp.han@siat.ac.cn (C. Han); lihf@sslab.org.cn (H. Li); cy.zhi@cityu.edu.hk (C. Zhi)

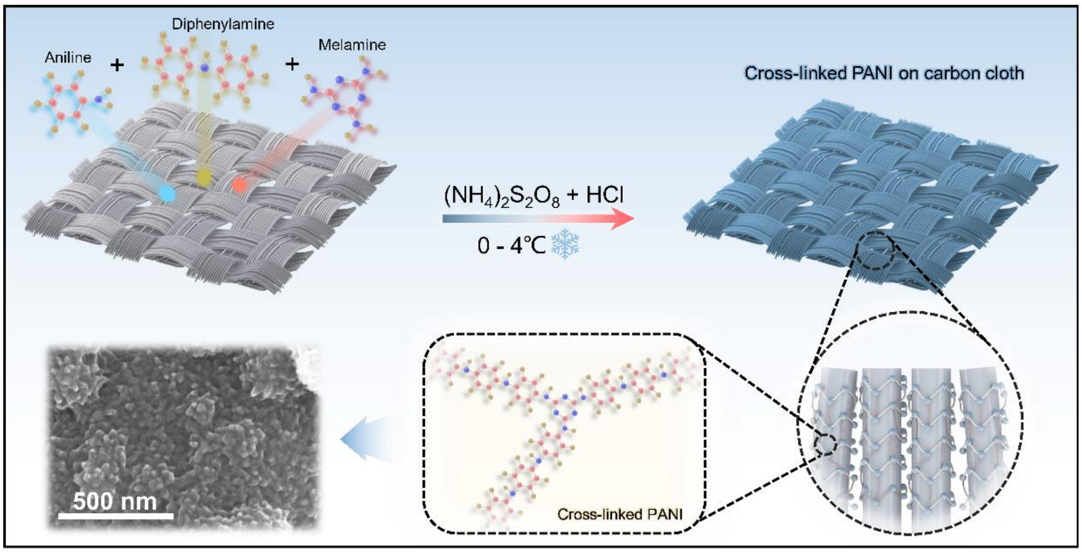

Supplementary Figure 1. Schematic synthesis of the C-PANI and SEM image of the C-PANI on the surface of carbon cloth.

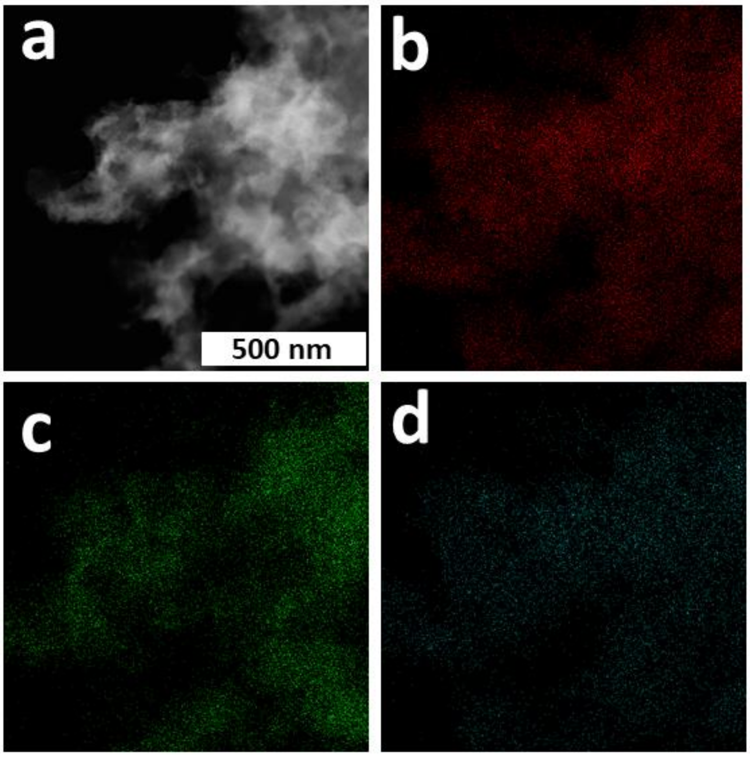

Supplementary Figure 2. The morphology and elemental distributions of C-PANI. TEM mages (a) and TEM-EDS mapping of C (b), Cl (c), and N (d) elemental distributions on C-PANI.

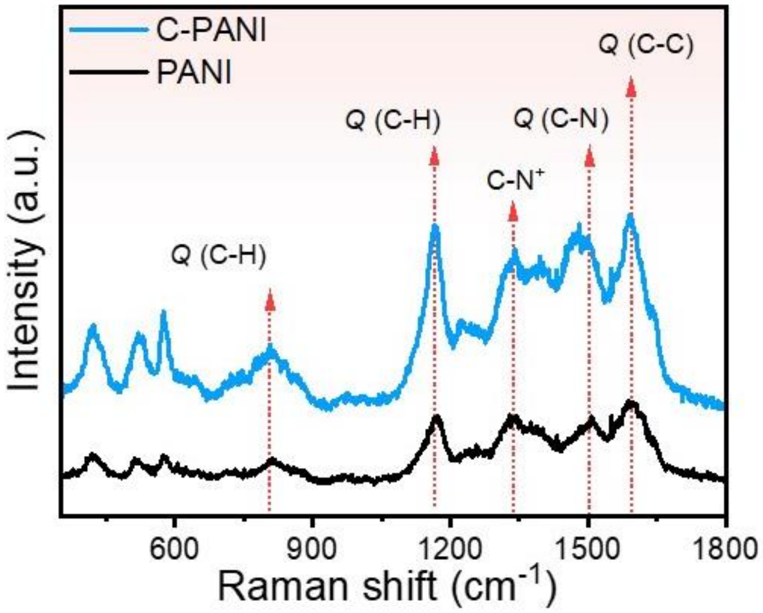

Supplementary Figure 3. Raman spectra using 633 nm lines. *Q* represents quinonoid ring-stretching vibrations. The test temperature is 25 °C± 1°C.

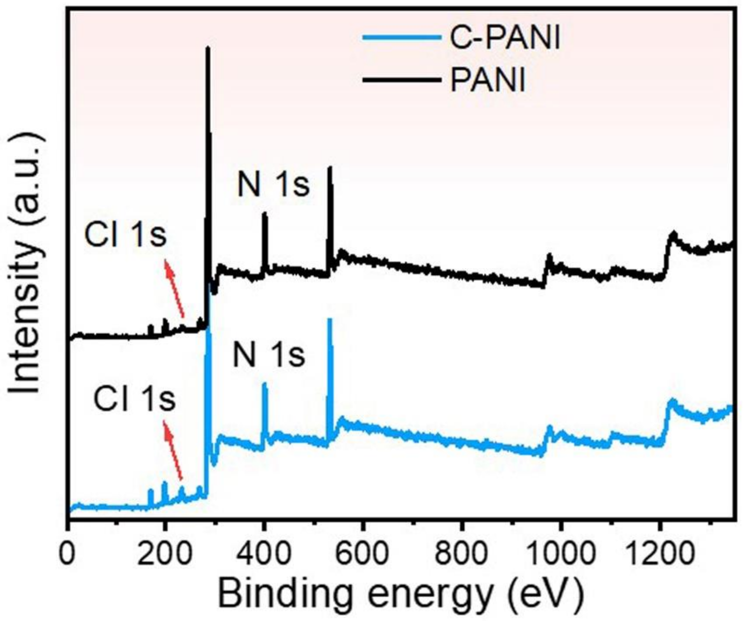

Supplementary Figure 4. XPS survey spectra of the samples.

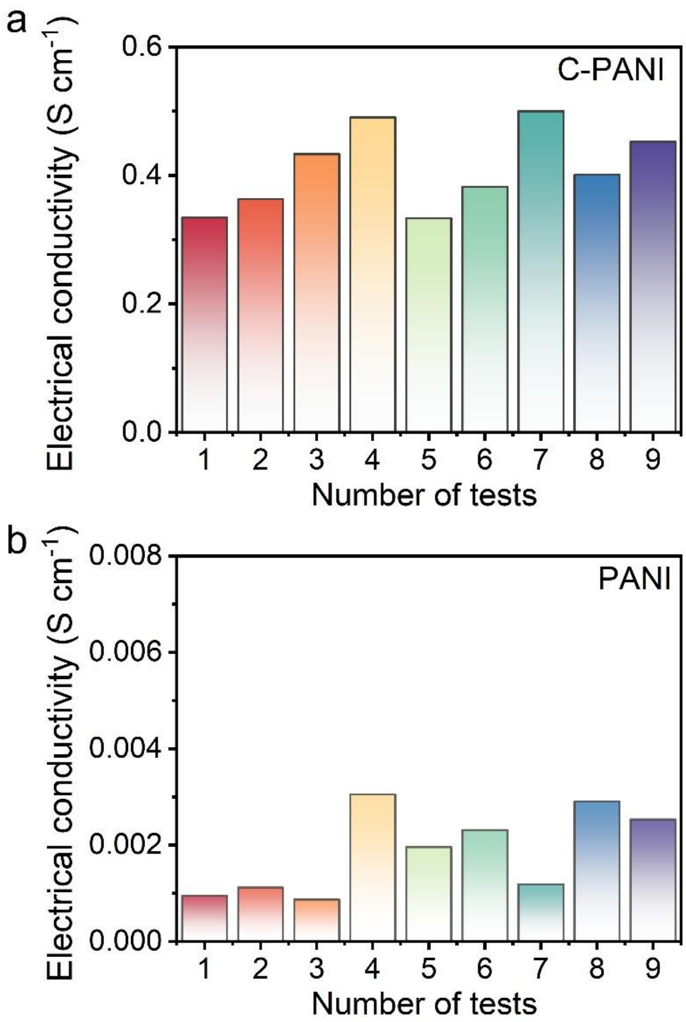

Supplementary Figure 5. The conductivities of samples. (a) C-PANI, and (b) PANI. The samples containing 0.3 g PANI or C-PANI were prepared in the infrared tablet press and tested in the four-probe test system. The test temperature is 25 °C± 1°C.

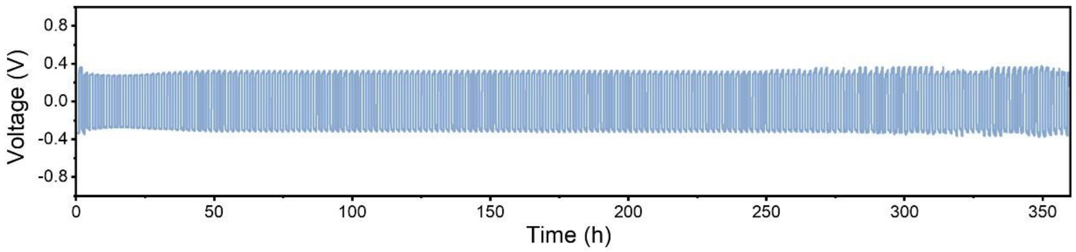

Supplementary Figure 6. Iron anode stability test. The voltage profiles of Fe||Fe coin cell at a current density of 1 mA cm^{-2} and capacity of 1 mAh cm^{-2} 1 M $Fe(TOF)_2$ electrolyte. The test temperature is 25 °C± 1°C.

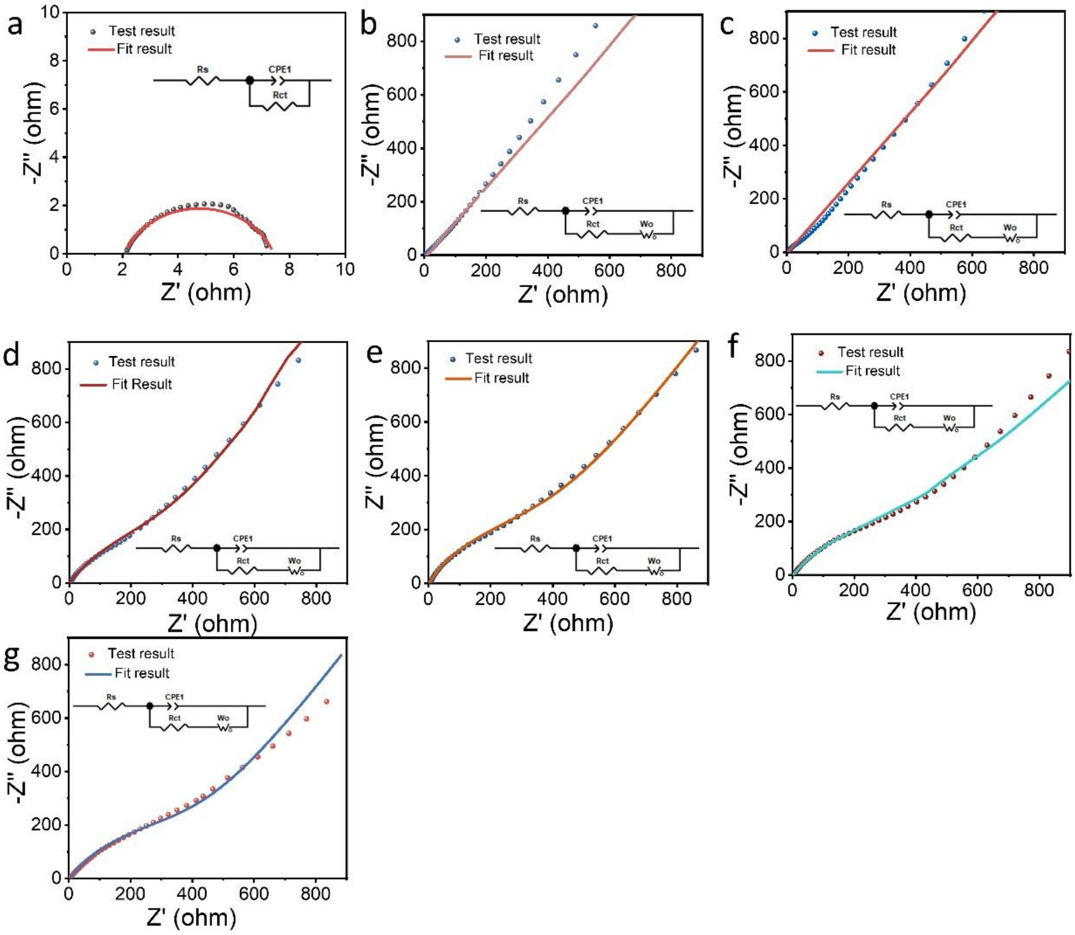

Supplementary Figure 7. EIS of the Fe||Fe symmetric coin cells at different cycles 1 M $Fe(TOF)_2$ electrolyte. The test temperature is 28 °C± 1°C. (a) 0 h, (b) 24 h, (c) 48 h, (d) 72 h, (e) 96 h, (f) 144 h, (g) 168 h. The corresponding equivalent circuit is used to simulate the resistances (inset): the ohmic resistance of solution and electrodes (R_I), the charge-transfer resistance (R_{CT}), the constant phase element (CPE), and the slope of the inclined line at flow frequencies corresponding to the Warburg resistance (W_o), respectively.

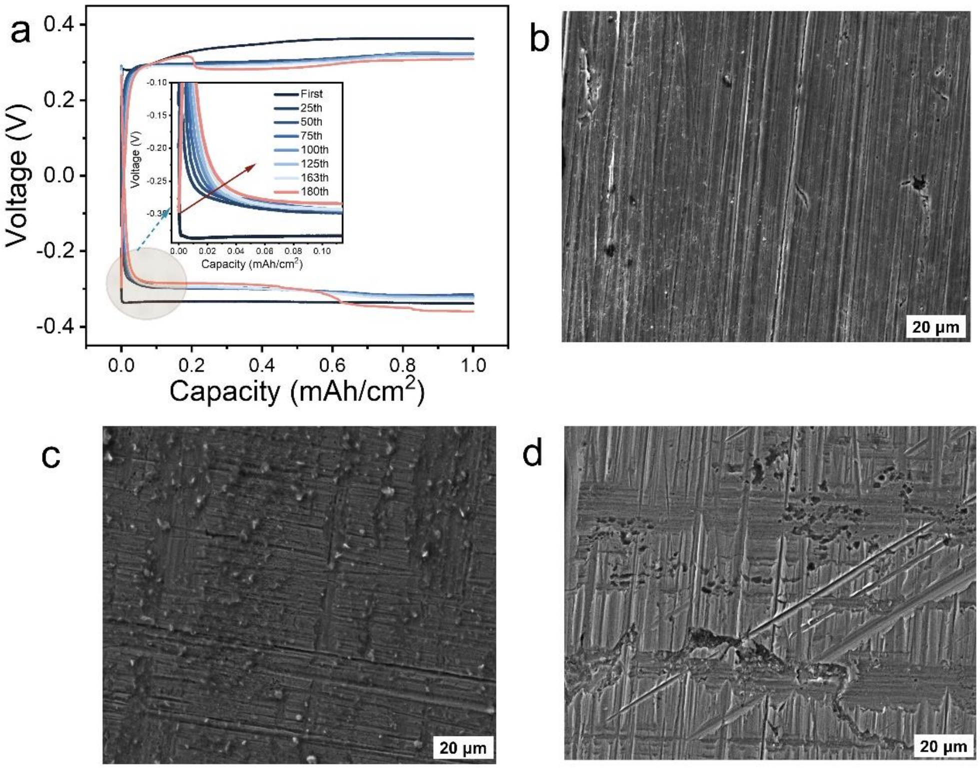

Supplementary Figure 8. Iron electrodes of Fe||Fe coin cell morphology and electrochemical characterization. (a) GCD plots of a Fe||Fe coin cell at different cycle numbers 1 M $Fe(TOF)_2$ electrolyte. (b) Fe electrode SEM before cycling. (c) Fe electrode after 1-hour electroplating. (d) Fe electrode after 1-hour stripping. The specific current was 1 mA cm^{-2} during cycling. The test temperature is 25 °C± 1°C.

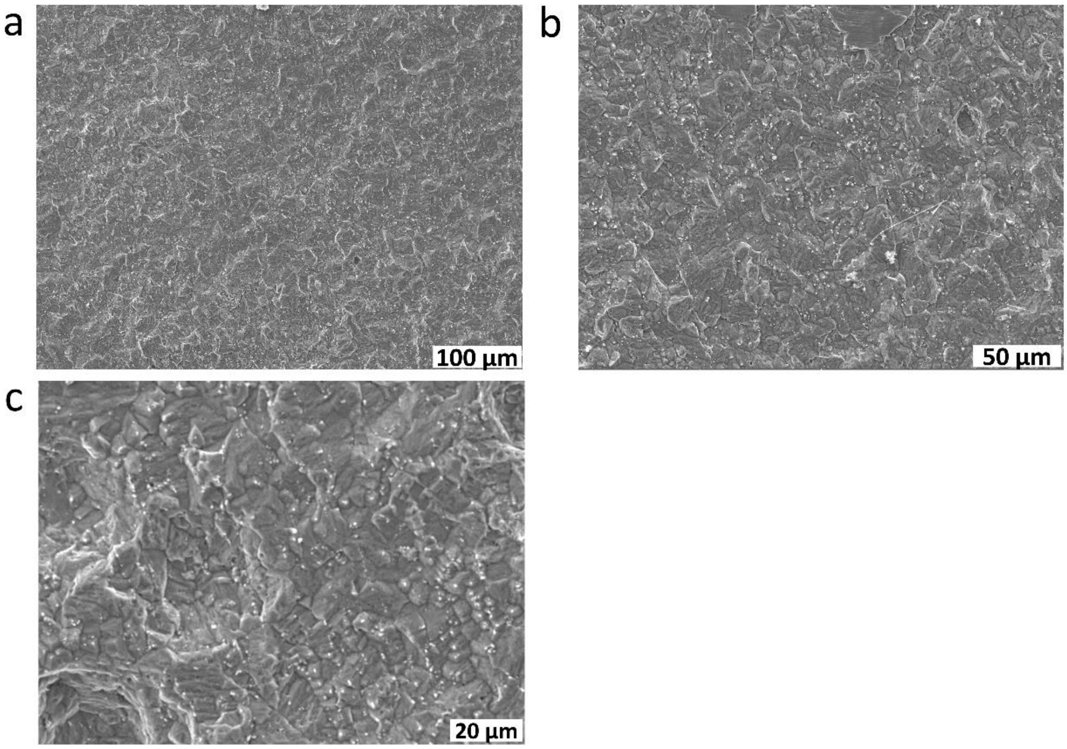

Supplementary Figure 9. SEM images at different magnifications of Fe electrode of symmetrical Fe||Fe coin cell after 168 hours cycling 1 M Fe(TOF)$_2$ electrolyte. (a) 1000 x, (b) 2000 x, (c) 4000 x. The specific current was 1 mA cm^{-2} during cycling. The test temperature is 25 °C± 1°C.

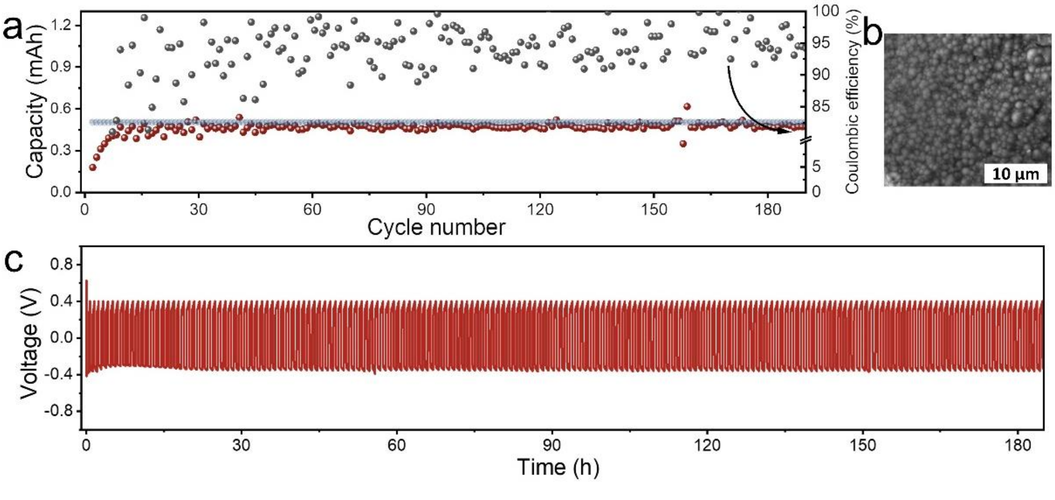

Supplementary Figure 10. Characterization of the iron-anode stability. (a) Coulombic efficiency of an asymmetrical Fe||Cu coin cell. (b) An SEM image of the plated Fe metal on copper foil for 1 hour. (c) Plating/stripping efficiency test. The specific current was 1 mA cm^{-2} during cycling. The test temperature is 28 °C± 1°C.

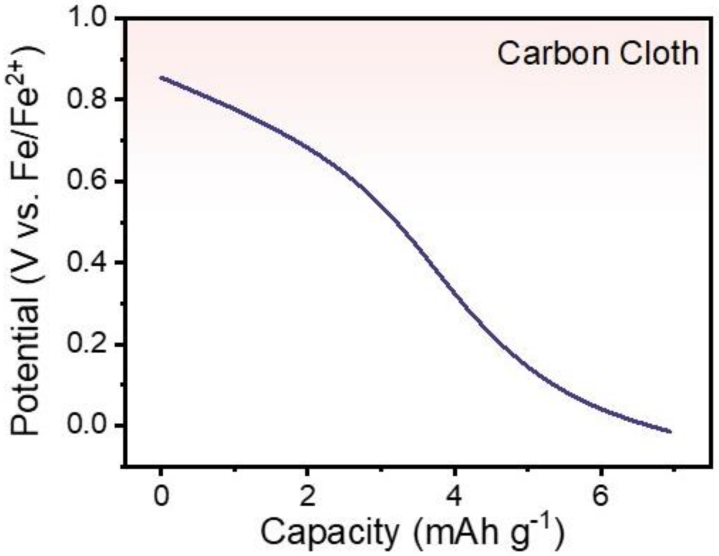

Supplementary Figure 11. Discharge profiles of the coin cell tested with carbon cloth serve as a cathode at 25 A g^{-1}. The test temperature is 28 °C± 1°C.

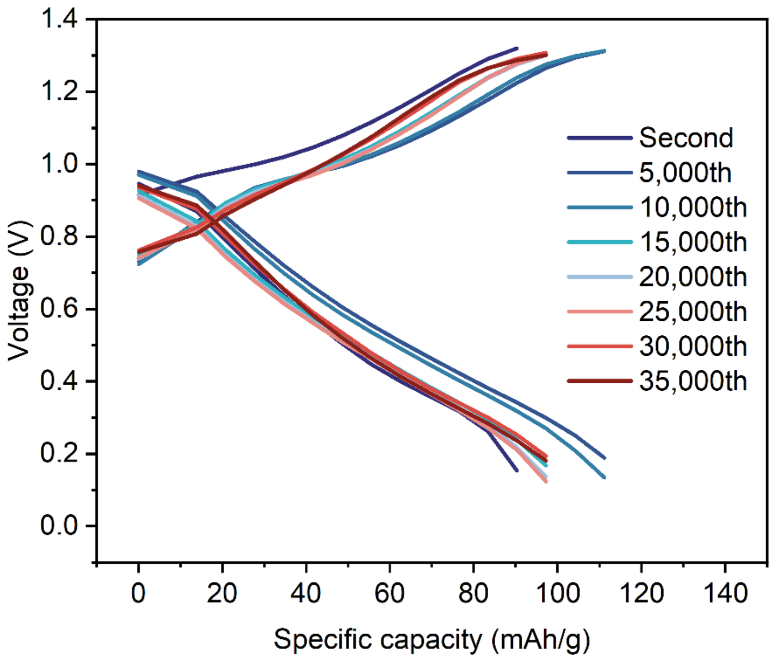

Supplementary Figure 12. The discharging-charging profiles of Fe||C-PANI based coin cell showing in Figure 2f. The specific current is 25 A g^{-1}. The test temperature is 28 °C± 1°C.

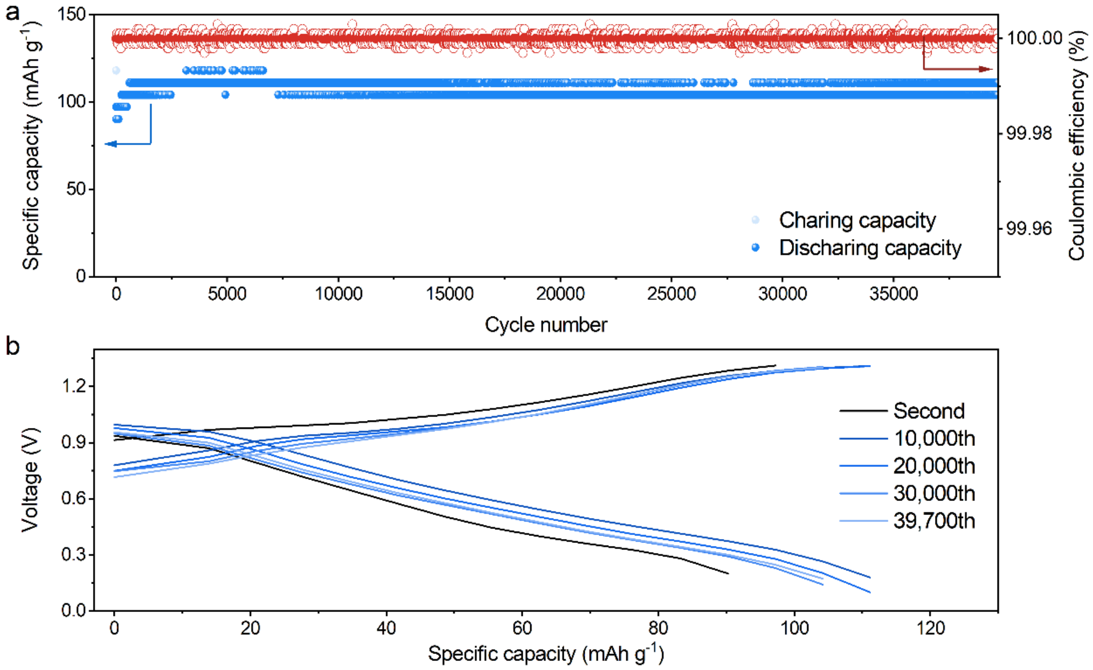

Supplementary Figure 13. Electrochemical characterization of another Fe||C-PANI coin cell. (a) The long-term cycling performance for another Fe||C-PANI coin cell (loading mass of ~1.1 mg cm^{-2}) at 25 A g^{-1}. (b) The discharging-charging profiles of the cell. The test temperature is 28 °C± 1 °C.

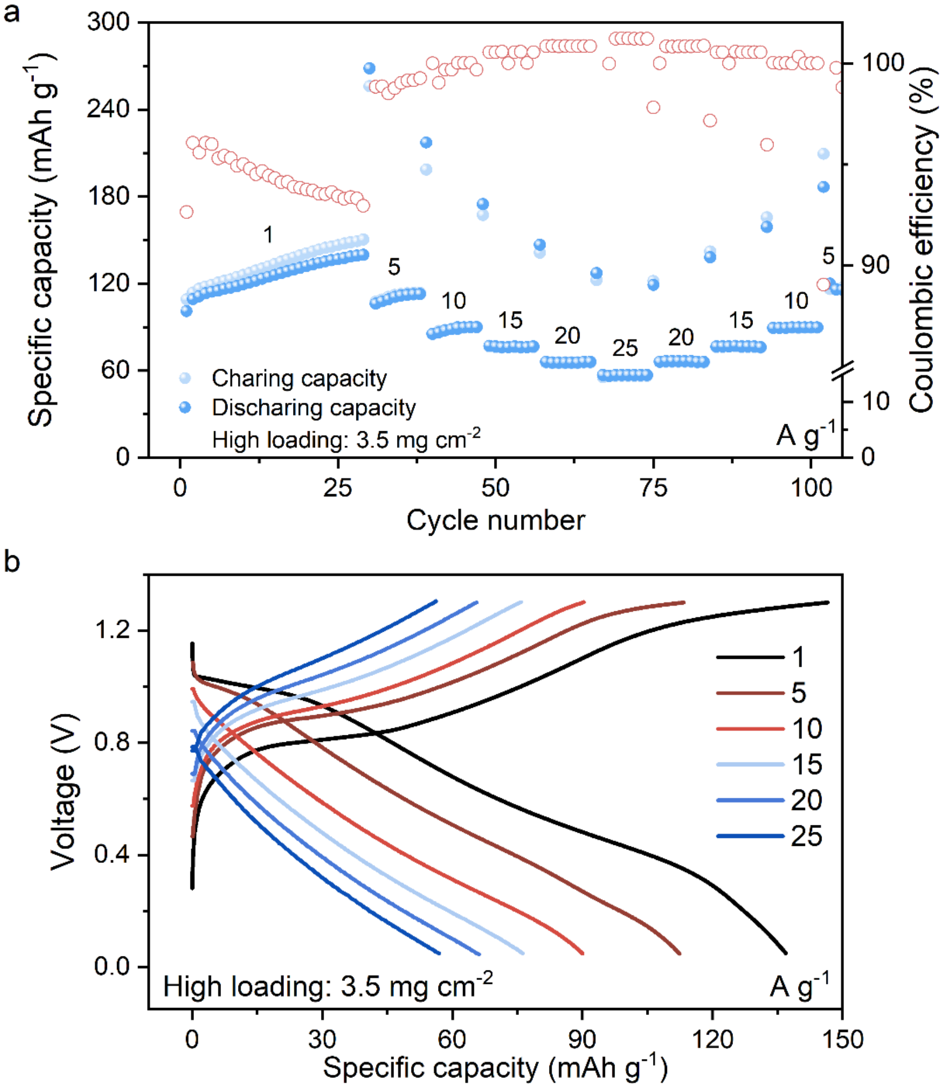

Supplementary Figure 14. Rate performance of high loading C-PANI. (a) Rate capability of Fe||C-PANI coin cells (loading mass of ~3.5 mg cm^2) at various specific currents from 1 to 25 A g^{-1} and (b) corresponding charge/discharge profiles. The test temperature is 28 °C± 1°C.

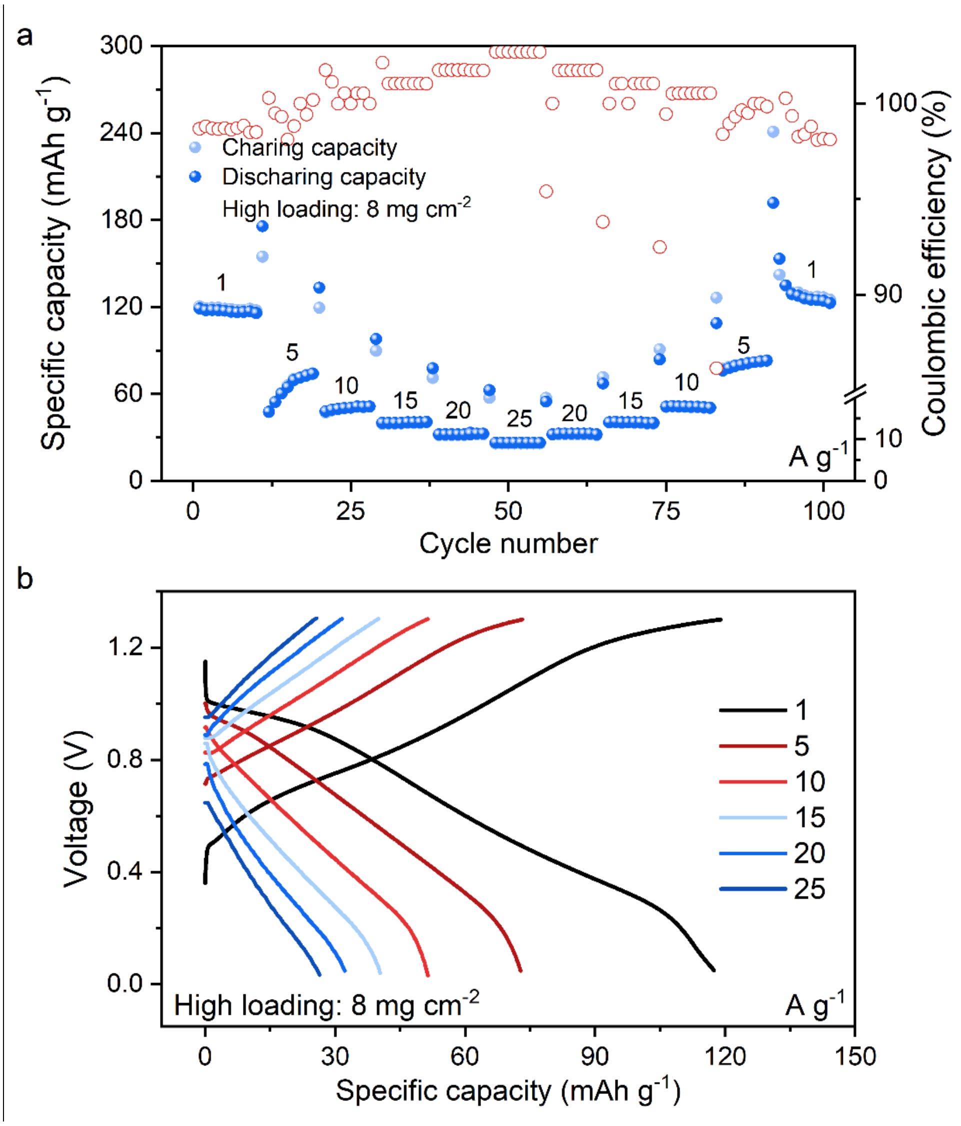

Supplementary Figure 15. Rate performance of high loading C-PANI. (a) Rate capability of Fe||C-PANI coin cells (loading mass of ~8 mg cm^2) at various specific currents from 1 to 25 A g^{-1} and (b) corresponding charge/discharge profiles. The test temperature is 28 °C± 1°C.

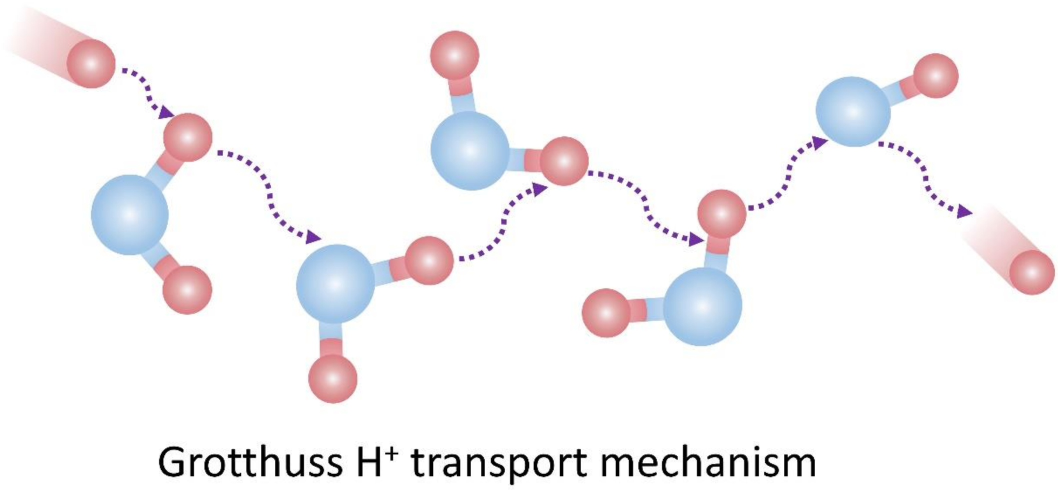

Supplementary Figure 16. Schematic diagram of the Grotthuss mechanism of proton. The red, blue spheres represent proton and oxygen, respectively. The purple dashed lines represent the conduction path of the proton.

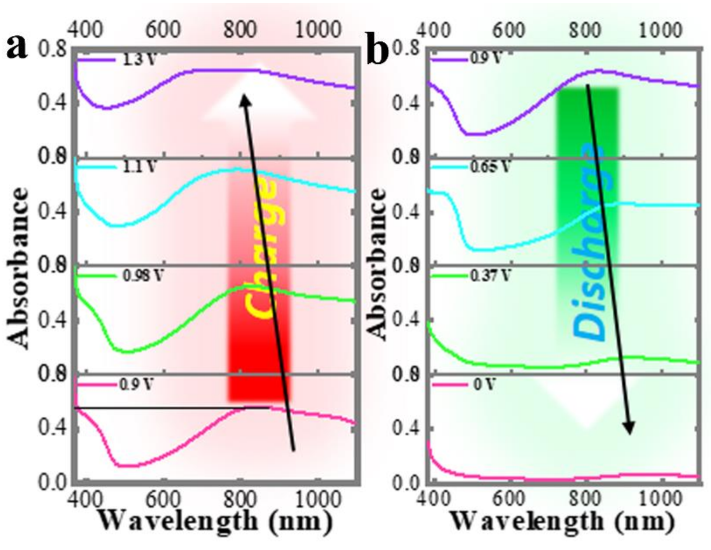

Supplementary Figure 17. In situ UV–vis of the C-PANI cathode of iron ion batteries in 1 M Fe(TOF)$_2$. (a) charging states and (b) discharging states. UV-vis spectra were achieved by C-PANI coated on the surface of conductive glasses as a working electrode. The test temperature is 28 °C± 1°C.

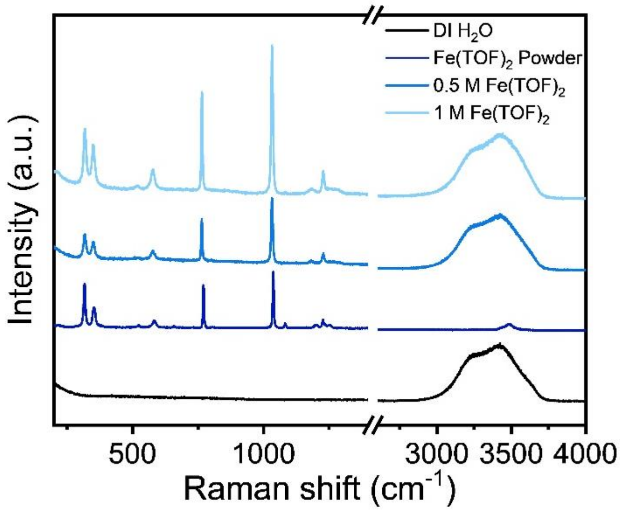

Supplementary Figure 18. Raman spectra of H_2O, $Fe(TOF)_2$ powder and electrolytes with different concentrations at an average temperature of 25 °C± 1°C.

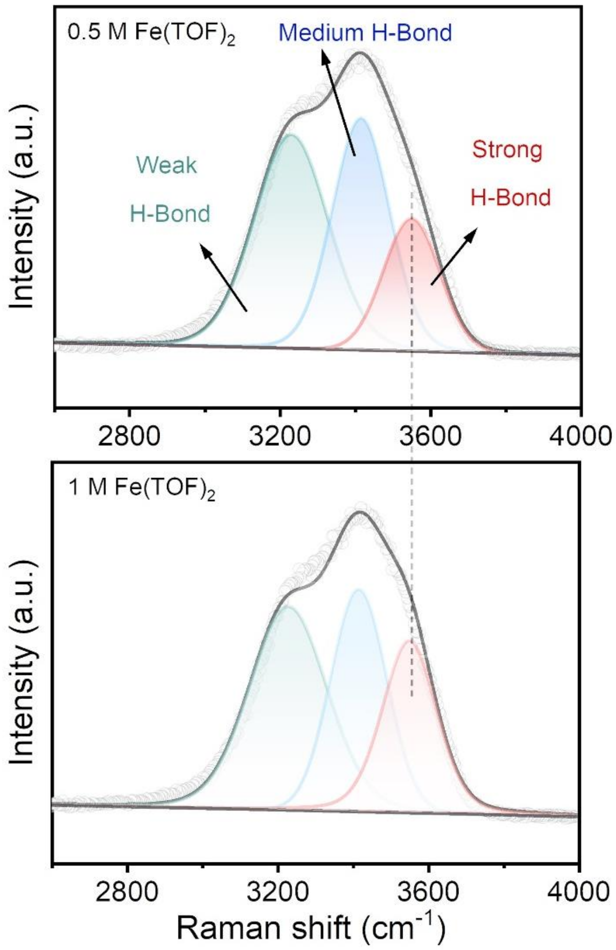

Supplementary Figure 19. Raman spectra of H-bonds in $Fe(TOF)_2$ solutions with different concentrations at an average temperature of 25 °C± 1°C.

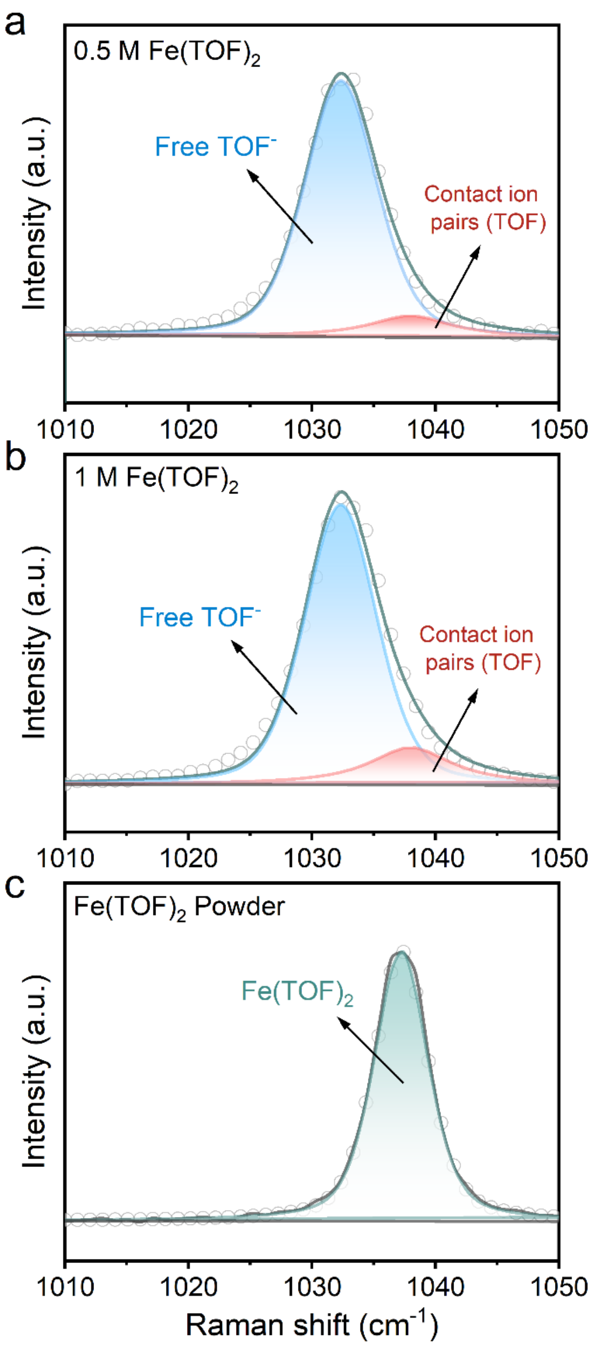

Supplementary Figure 20. Raman spectra of O=S bond of Fe(TOF)$_2$ electrolytes with different concentrations and Fe(TOF)$_2$ powder at an average temperature of 25 °C± 1°C. (a) 0.5 M Fe(TOF)$_2$, (b) 1 M Fe(TOF)$_2$, and (c) Fe(TOF)$_2$ Powder.

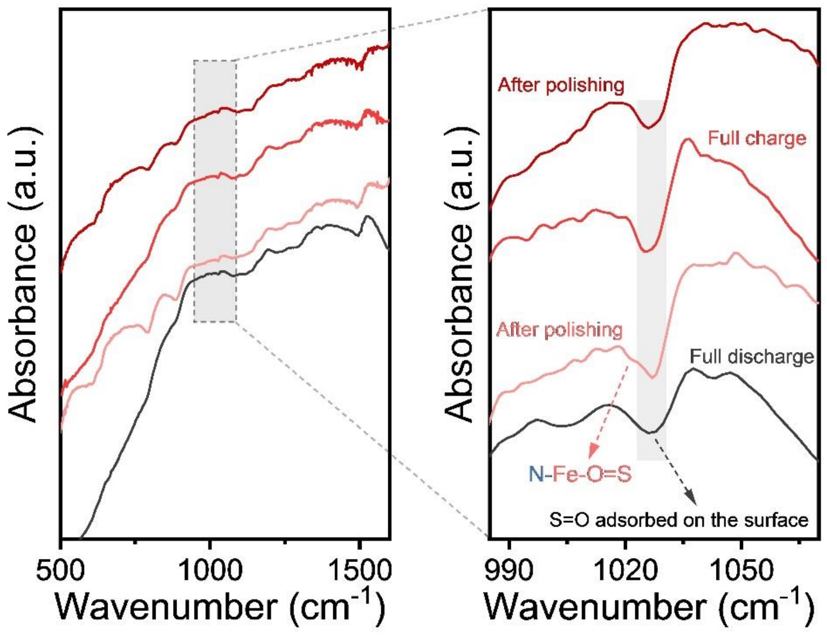

Supplementary Figure 21. Ex situ FT-IR spectra before and after polishing the C-PANI electrodes at full discharge/full charge at an average temperature of 25 °C± 1°C.

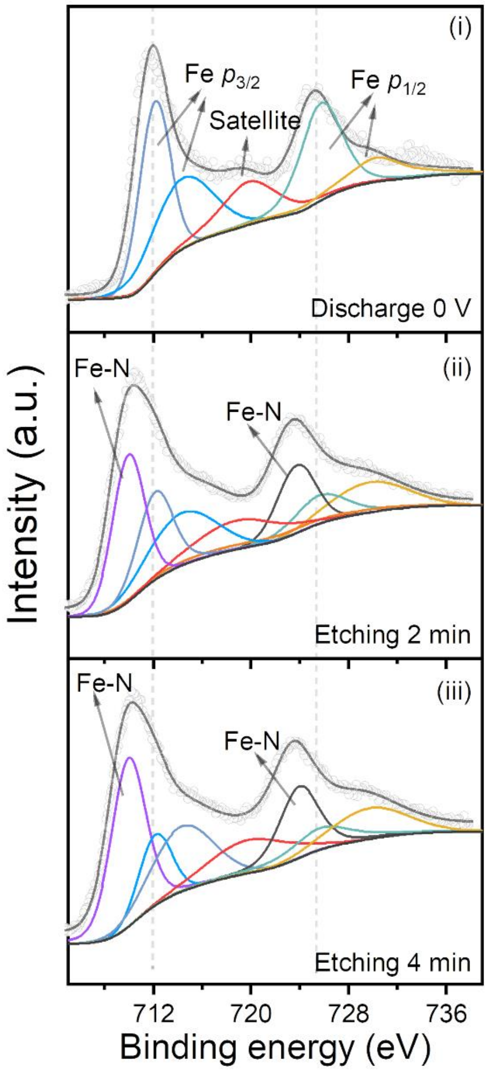

Supplementary Figure 22. Ex situ XPS spectra of Fe 2p of C-PANI cathodes were obtained at a fully discharge state in different etching time. The test temperature is 25 °C± 1°C.

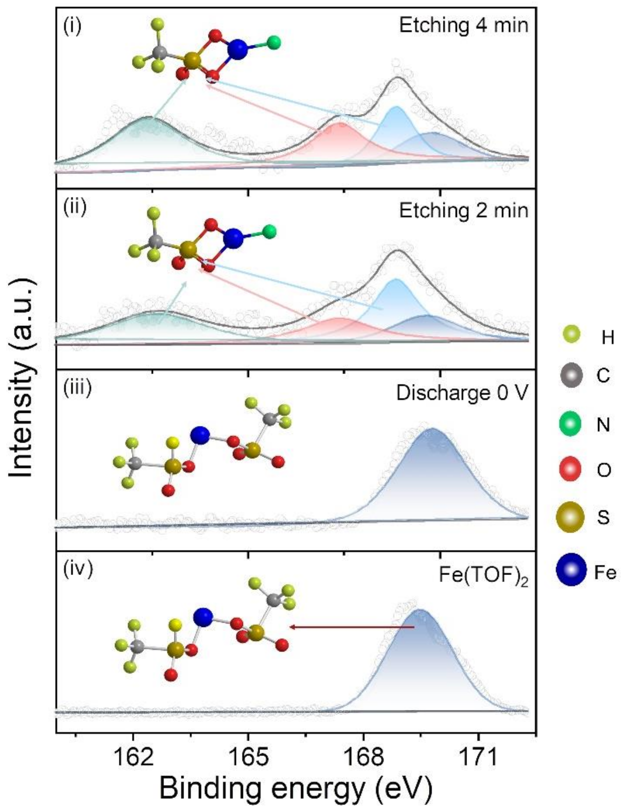

Supplementary Figure 23. Ex situ XPS spectra of S 2*p* of C-PANI cathodes obtained at a fully discharge state in different etching time and Fe(TOF)₂. The test temperature is 25 °C± 1°C.

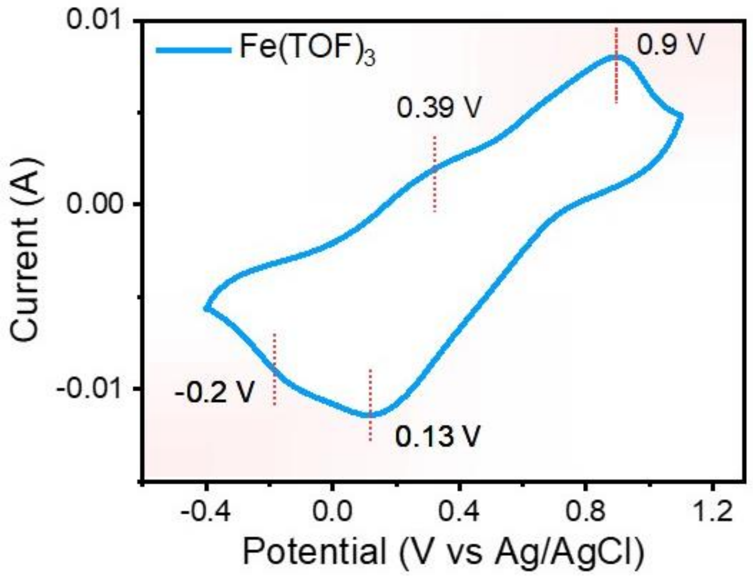

Supplementary Figure 24. CV curves of C-PANI in 0.1 M Fe(CF_3SO_3)$_3$ electrolyte measured by three-electrode systems at 2 mV s^{-1} in Glass cell with an average temperature of 28 °C± 1°C.

.

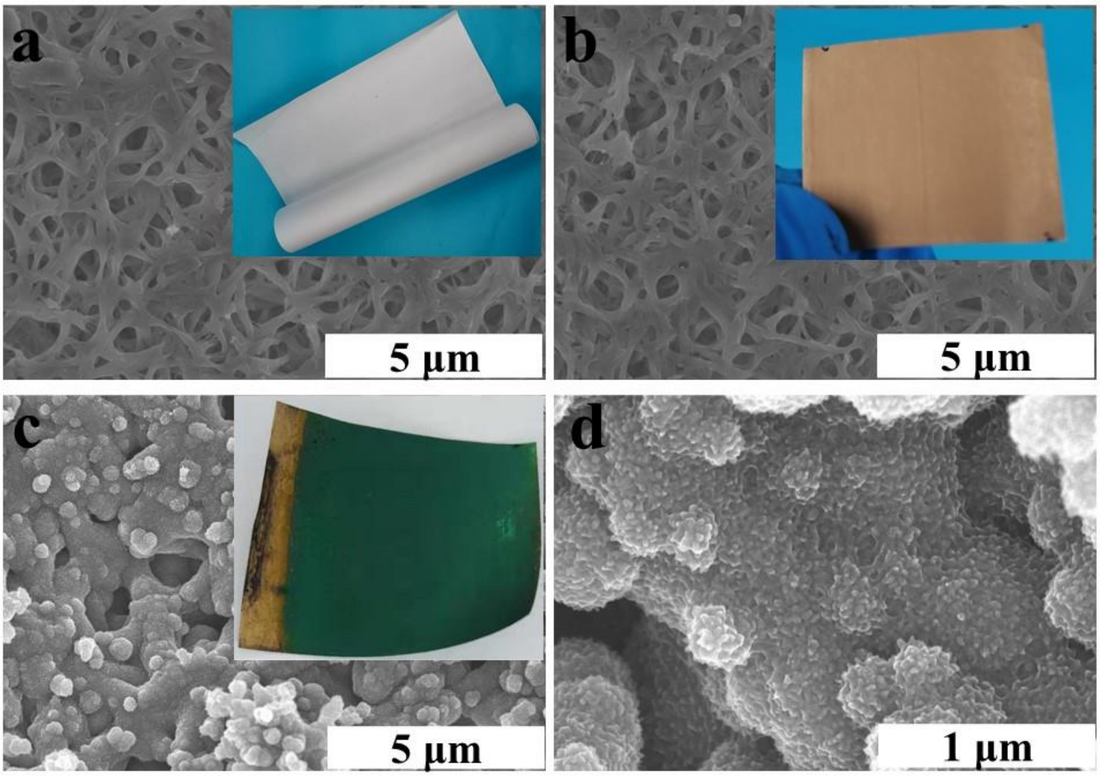

Supplementary Figure 25. Cathode morphology characterisation of flexible reflective electrochromic batteries. (a) SEM image of nylon 66. The inset shows an optical photo of nylon 66. (b) SEM image of Au-coated nylon 66 and the corresponding optical photo. (c), (d) C-PANI on Au-coated nylon 66.

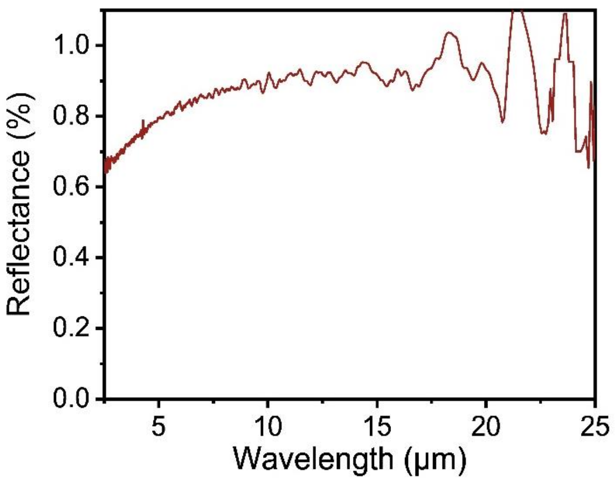

Supplementary Figure 26. **Specular FTIR diffuse reflectance of Au-coated nylon 66 was conducted at an average temperature of 25 °C± 1°C.**

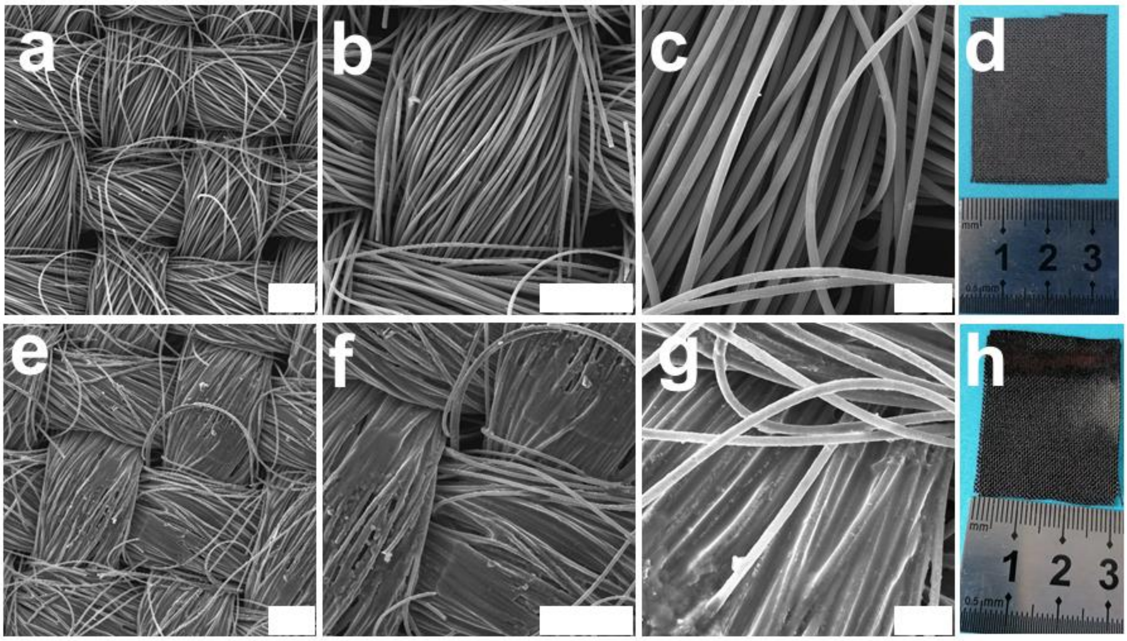

Supplementary Figure 27. SEM image of carbon cloth. (a) Scale bars 200 µm, (b) Scale bars 200 µm, (c) Scale bars 50 µm. (d) optical photo of carbon cloth. SEM image of Fe on carbon cloth by electrodeposition in 1 M $Fe(ClO_4)_2$. (e) Scale bars 200 µm, (f) Scale bars 200 µm, (g) Scale bars 50 µm, (h) optical photo of Fe carbon cloth. The SEM image shows that the electroplated Fe on the carbon cloth exists as filling carbon fiber.

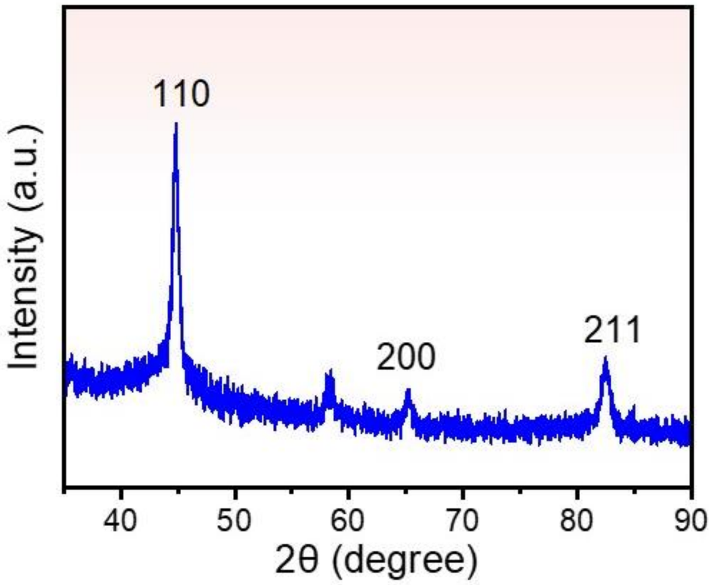

Supplementary Figure 28. XRD patterns of Fe on carbon cloth by electrodeposition. The XRD patterns show the crystalline phases of the electroplated Fe metal in 1M $Fe(ClO_4)_2$. The diffraction peaks observed at 44.8, 65.2 and 82.5 can be assigned to the (110), (200) and (211) plane of Fe, which are well indexed to Fe (JCPDS: 06-0696). This indicates that the as-prepared Fe is highly crystalline.

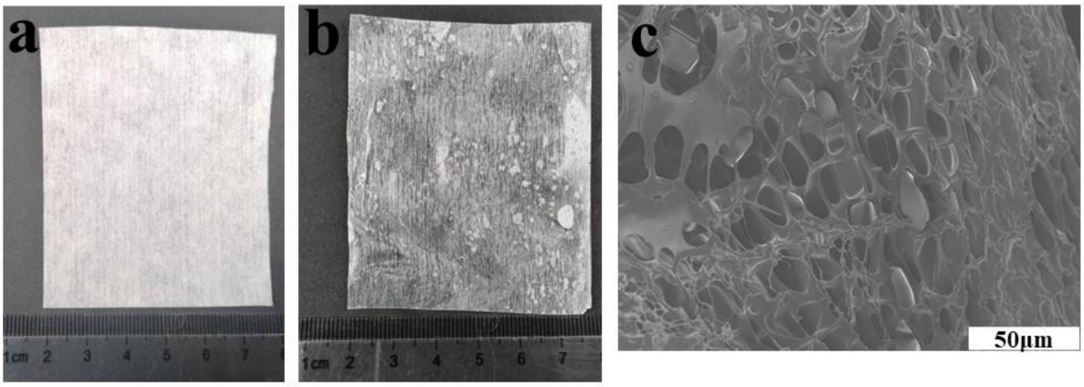

Supplementary Figure 29. The optical photo. (a) non-woven cloth, (b) hydrogel and non-woven cloth composite film, (c) SEM image of the composite film.

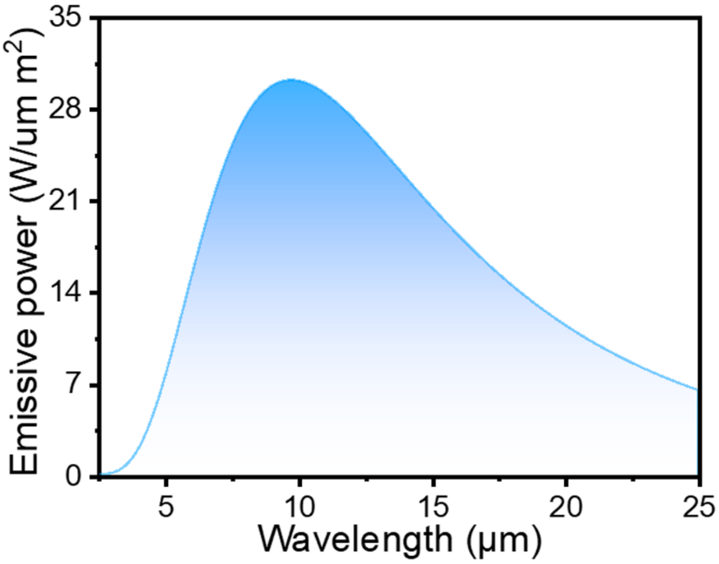

Supplementary Figure 30. The ideal black body spectra (298.15 K). The ideal black body spectrum is a slightly asymmetric bell shaped curve with a long wavelength tail.

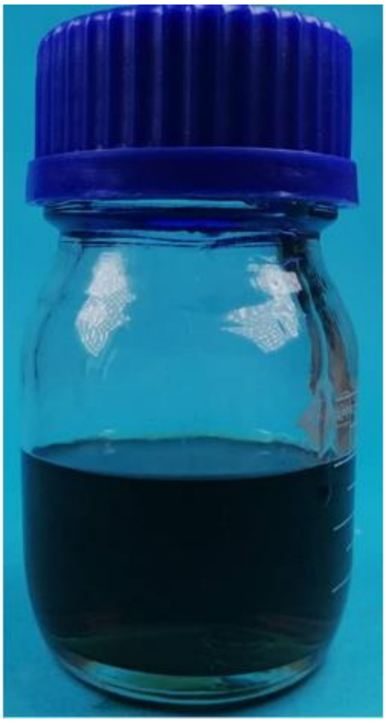

Supplementary Figure 31. The optical photo of 1 M $Fe(ClO_4)_2$ in propylene carbonate.

Supplementary table 1. Comparison of our Fe||Cross-linked PANI battery with the reported energy storage devices in terms of cycle numbers, flexible, electrochromic, maximum applied current, and short circuit warning.

Ref	Cell configuration	Cycle numbers	Flexible	Electrochromic	The maximum applied current	Hydrogel electrolyte	Testing temperatures
3	Fe||S	200	no	no	Not provided	no	Not provided
4	Fe||Prussian blue analogue cathode	1000	no	no	2.4 A g^{-1}	no	Not provided
5	Fe||I_2	550	no	no	2 A/g	no	Room temperature
6	Zn||PANI	1000	yes	yes	10 A/g	no	Not provided
7	Zn||Mo/Ti:WO_3	100	no	yes	Not provided	no	Room temperature
8	Zn||WO_3		no	yes	Not provided	no	Room temperature
9	Zn||Polypyrrole	50	no	yes	Not provided	no	Not provided
10	Zn||Co_3O_4	5000	yes	no	8 A/g	yes	Not provided
11	Zn|| Prussian blue analogue	10000	yes	no	8 A/g	yes	25 °C
12	Al||Polypyrrole	50	no	yes	2 A/g	no	Not provided
13	Zn||$Fe_4^{III}[Fe^{II}(CN)_6]_3$)	2000	no	yes	0.7 A/m^2	no	Room temperature
14	Zn||Co_3O_4	6000	yes	no	3.75 A/g	no	Not provided
**This work**	**Fe||C-PANI**	**27 000**	**yes**	**yes**	**25 A/g**	**yes**	**28 °C± 1°C**

Supplementary table 2. The R_{CT} values of the Fe||Fe symmetric coin cells in equivalent circuit models for EIS with the stripping/plating cycling measurements for various times.

	The stripping/plating cycling measurements for various times						
Time	0 h	24 h	48 h	72 h	96 h	144 h	168 h
R_{CT} (ohm)	5.36±0.07	2.958±0.11	161.5±9.98	603.4±54.3	610±41.48	659.3±41.4	642.6±61.68

Supplementary note 1

Stability study of the symmetrical Fe||Fe coin cell

We evaluate the symmetric cells for stability study. The charge/discharge tests (Supplementary Figure 6) were conducted under a current density of 1 mA/cm^2. The potential polarization is over ~ 640 mV and the cell can run steadily over 360 h without evident potential fluctuations. The evolution of the impedance results of the symmetric cells revealed their electrochemical stability (Supplementary Figure 7). The charge transfer resistance (Rct) of the cell first increased, and kept steady after 96 hours (Supplementary table 2); such large variations in Rct of the cell indicate the enlarged surface areas and other inactive products during the cycled process. The potential polarization of the first cycle (0.69 V) is greater than that of the following cycles, due to higher nucleation overpotential in the initial interval (Supplementary Figure 8). Furthermore, the Fe electrode morphologies of plating and tripping were further investigated after the cell was cycled 1 hour at 1 mA h cm^{-2} (Supplementary Figure 8c and 8d). The plated Fe exhibited nonuniform Fe morphology with small Fe granules. The striped Fe shows the shape of pits and concave (Supplementary Figure 8c and 8d). After 168 hours, the surface of the plated iron is dense and composed of large particles of a few micrometers in size under scanning electron microscopy (Supplementary Figure 9). The morphology is beneficial for improving the stability of the battery.[2]

Supplementary note 2

Asymmetrical Fe||Cu cells study

To check the Fe chemical stability, the Coulombic efficiency of metal plating/stripping was conducted in asymmetrical Fe||Cu cells. As shown in Supplementary Figure 10, The Coulombic efficiency is ≈37.2% in the first cycle, possibly attributed to unstable anode interface reactions like passivation layer fracture or reformation, and then the Coulombic efficiency values reach ~ 100% in subsequent cycles. Additionally, the morphology of iron on copper foil deposited for 1 hour was investigated simultaneously. The plated iron exhibited smooth submicrometer-sized particles and was compactly stacked, which potentially benefits battery stability (Supplementary Figure 10b).

Supplementary references

(1) Zhang, Q. *et al.* Chaotropic Anion and Fast-Kinetics Cathode Enabling Low-Temperature Aqueous Zn Batteries. *ACS Energy Lett.* 6, 2704−2712 (2021)

(2) Du, W. Yan, J. Cao, C & Li, C. Electrocrystallization orientation regulation of zinc metal anodes: strategies and challenges. *Energy Storage Mater.* 52, 329-354 (2022)

(3) Wu, X. *et al.* Rechargeable Iron–Sulfur Battery without Polysulfide Shuttling. *Adv. Energy Mater.* 9, 1902422 (2019)

(4) Wu, X. *et al.* A Rechargeable Battery with an Iron Metal Anode. *Adv. Funct. Mater.* 29, 1900911 (2019).

(5) Bai, C. Jin, H. Gong, Z. Liu, X. & Yuan, Z. A high-power aqueous rechargeable Fe-I_2 battery. *Energy Storage Mater. 28*, 247-254 (2020).

(6) Wang, Y. Jiang, H.; Zheng, R.; Pan, J.; Niu, J.; Zou, X.; Jia, C. A flexible, electrochromic, rechargeable Zn-ion battery based on actiniae-like self-doped polyaniline cathode. *J. Mater. Chem. A* 8, 12799-12809 (2020).

(7) Li, H. *et al.* Rechargeable Aqueous Electrochromic Batteries Utilizing Ti-Substituted Tungsten Molybdenum Oxide Based Zn(2+) Ion Intercalation Cathodes. *Adv. Mater.* 31, e1807065 (2019).

(8) Li, H., Firby, C. J., Elezzabi, A. Y. Rechargeable Aqueous Hybrid Zn^{2+}/Al^{3+} Electrochromic Batteries. *Joule* 3, 2268-2278 (2019).

(9) Wang, J. *et al.* A flexible, electrochromic, rechargeable Zn||PPy battery with a short circuit chromatic warning function. *J. Mater. Chem. A 6*, 11113-11118 (2018).

(10) Ma, L. *et al.* Initiating a mild aqueous electrolyte Co_3O_4/Zn battery with 2.2 V-high voltage and 5000-cycle lifespan by a Co(iii) rich-electrode. *Energy Environ. Sci.*11, 2521-2530 (2018).

(11) Yang, Q. *et al.* Activating C-Coordinated Iron of Iron Hexacyanoferrate for Zn Hybrid-Ion Batteries with 10 000-Cycle Lifespan and Superior Rate Capability. *Adv. Mater.* 31, 1901521 (2019).

(12) Yang, B. *et al.* A self-rechargeable electrochromic battery based on electrodeposited polypyrrole film. *Sol. Energy Mater. Sol. Cells* 192, 1-7 (2019).

(13) Wang, B.; Cui, M. W.; Gao, Y. F.; Jiang, F. Y.; Du, W.; Gao, F.; Kang, L. T.; Zhi, C. Y.; Luo, H. J. A Long-Life Battery-Type Electrochromic Window with Remarkable Energy Storage Ability. *Sol. RRL* 4, 1900425 (2020).

(14) Qun, G. *et al.* Dendrite-Free Flexible Fiber-Shaped Zn Battery with Long Cycle Life in Water and Air. *Adv. Energy Mater.* 9, 1901434 (2019).
